# High-resolution cortical parcellation based on conserved brain landmarks for localization of multimodal data to the nearest centimeter

**DOI:** 10.1038/s41598-022-21543-3

**Published:** 2022-11-05

**Authors:** Hari McGrath, Hitten P. Zaveri, Evan Collins, Tamara Jafar, Omar Chishti, Sami Obaid, Alexander Ksendzovsky, Kun Wu, Xenophon Papademetris, Dennis D. Spencer

**Affiliations:** 1 Department of Neurosurgery, Yale School of Medicine, New Haven, CT USA; 2 GKT School of Medical Education, King’s College London, London, UK; 3 Department of Neurology, Yale School of Medicine, New Haven, CT USA; 4 Yale School of Engineering and Applied Science, New Haven, CT USA; 5 Department of Biological Engineering, Massachusetts Institute of Technology, Cambridge, MA USA; 6 Department of Neurosurgery, University of Maryland School of Medicine, Baltimore, MD USA; 7 Department of Radiology and Biomedical Engineering, Yale School of Medicine, New Haven, CT USA; 8 Department of Biomedical Engineering, Yale School of Medicine, New Haven, CT USA

**Keywords:** Computational neuroscience, Neuroscience, Anatomy, Neurology

## Abstract

Precise cortical brain localization presents an important challenge in the literature. Brain atlases provide data-guided parcellation based on functional and structural brain metrics, and each atlas has its own unique benefits for localization. We offer a parcellation guided by intracranial electroencephalography, a technique which has historically provided pioneering advances in our understanding of brain structure–function relationships. We used a consensus boundary mapping approach combining anatomical designations in Duvernoy’s Atlas of the Human Brain, a widely recognized textbook of human brain anatomy, with the anatomy of the MNI152 template and the magnetic resonance imaging scans of an epilepsy surgery cohort. The Yale Brain Atlas consists of 690 one-square centimeter parcels based around conserved anatomical features and each with a unique identifier to communicate anatomically unambiguous localization. We report on the methodology we used to create the Atlas along with the findings of a neuroimaging study assessing the accuracy and clinical usefulness of cortical localization using the Atlas. We also share our vision for the Atlas as a tool in the clinical and research neurosciences, where it may facilitate precise localization of data on the cortex, accurate description of anatomical locations, and modern data science approaches using standardized brain regions.

## Introduction

Brain atlas mapping is a method for localizing data in a common space according to biomarkers of brain structure and function^1,2^. One example is the HCP-MMP1 (Glasser) atlas, where the authors used multiple imaging modalities along with a semi-automated parcellation algorithm to capture four cortical properties which derived the parcellation: architecture, function, connectivity, and topography^3^. Several widely-used atlases have used similarly robust methods, including the Human Brainnetome Atlas^4^, the Yeo Atlas^5^, and the Schaefer Atlas^6^.

Many studies, particularly those in the clinical sciences, require precise cortical localization to map the topographic location of features in the brain. Some studies then link this localization to previously identified connectomic, functional, or architectonic patterns, while other studies report solely on the topographic localization; for instance, studies mapping cortical function based on surgical data^7–9^, studies mapping seizure onset and spread in epilepsy^10,11^, and studies mapping the anatomical distribution of brain tumors^12,13^. Precise cortical localization poses a major challenge in atlas mapping due to inter-individual anatomical variability, which necessitates larger parcels to ensure that atlases can fit common anatomy. This limits the resolution of many atlases^1^. Prior atlases have been created to optimize cortical localization, including the Destrieux atlas of common neuroanatomy and the Talairach stereotaxic atlas^14–17^. An atlas that combines the anatomical cortical accuracy of the Destrieux atlas and others with the high-resolution coordinate system concept of the Talairach atlas may be valuable.

Parcellated brain atlases are rarely used directly in clinical workflows or in education workflows outside of research studies, which may be due in-part to a lack of standardized brain regions between atlases, and inaccuracies in image registration leading to errors in atlas localization^18^. In many disciplines this underutilization may be due to the need to retain a high-resolution localization of the data in individual subject space. This precludes the dimension reduction of atlases where we cluster brain localization data into manageable parcels, often in a common anatomical space. In epilepsy surgery and neuro-oncology, data from electrophysiology, functional mapping and cortical-cortical evoked potentials is procured at the resolution of a centimeter of cortex using intracranial electroencephalography (icEEG) electrodes with contacts spaced one centimeter apart^19^. It is important to retain the native spatial resolution of icEEG signals and the information which is captured by them to facilitate high-quality clinical and research work. There is therefore a need for an atlas that offers centimeter-level cortical parcellation with anatomical segregation and nomenclature. An atlas offering this level of precision could be extended to any field of neuroscience where systematic, sub-gyral cortical localization is desirable, including where high-resolution imaging modalities are used thus allowing multi-modal data fusion.

In this study we attempt to address these pertinent issues in brain atlas mapping with a new atlas, the Yale Brain Atlas (YBA), and nomenclature, based on decades of work in the surgical treatment of epilepsy. We address individual anatomical variation by parcellating discrete regions of the brain according to a limited number of reproducible, conserved neuroanatomical landmarks. To address limited cortical resolution, we have parcellated these regions into approximately one-square centimeter sections while respecting local anatomical features. The one-square centimeter parcellation respects the native acquired resolution of surgical epilepsy and oncology data, where electrode contacts are typically spaced one centimeter apart to record high resolution brain data in-vivo. Further, the one-square centimeter parcellation provides a rich subdivision of sub-lobar areas while maintaining accurate brain image registration, which may be compromised by finer parcellation. This parcellation may also provide real-world applicability for communication in research and clinical practice. Finally, we offer an anatomical labeling system for communicating and correlating these regions of interest. To test whether our one-square centimeter parcellation is appropriate, we evaluate the accuracy of parcel-level image registration using intracranial electrode contacts as an anatomical localization substrate. We also provide the results of a cohort atlas localization study, where our goal is to precisely localize and display many intracranial electrode contacts across a cohort of patients on a single reference atlas. We propose this atlas as a visual substrate for the correlation of multimodal data, including imaging and electrophysiology, across many individuals.

## Results

### Atlas features

**Figure 1:**
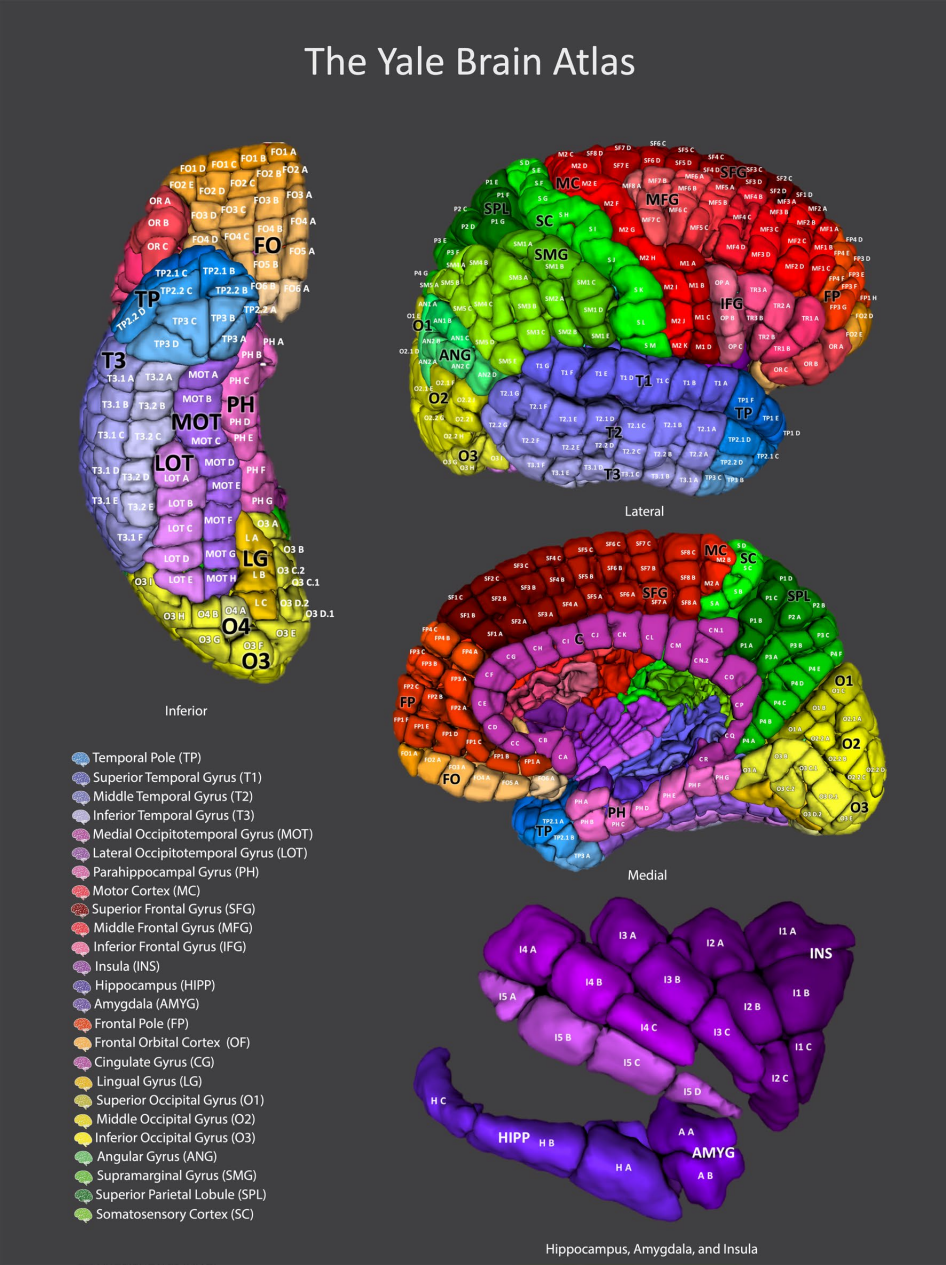
A color illustration of the Yale Brain Atlas with each surface depicted and parcels labelled using the Atlas nomenclature. Large, regional labels (black) are provided which link to common anatomical nomenclature in the key. The Atlas consists of 690 parcels and 144 gyri across the cortex. Here the surfaces of the right hemisphere of the Atlas are provided for illustration. The left hemisphere is a mirror image of the right and contains the same parcels. Each gyrus is defined based on its proximity to conserved neuroanatomical landmarks. The parcels are standardized to a length of approximately one centimeter along the gyrus and are named sequentially with a gyrus code and a letter indicating their position within the gyrus.

The Yale Brain Atlas (YBA) is a whole-brain atlas of the cortex, hippocampus, and amygdala with 690 parcels and 144 gyri within 34 discrete, sub-lobar regions, including the temporal pole and frontal orbital cortex, and 10 lobes. Each parcel has a unique code, identifying the gyrus and the centimeter increment where it lies along the gyrus. A full list of the gyri and parcels is provided as Supplementary Table 1 in Supplementary Material. A visual reference is provided in Fig. 1 with labelled parcels. The YBA can be downloaded from https://www.nitrc.org/projects/yale_atlas_2021/ and it can be viewed at https://yalebrainatlas.github.io/YaleBrainAtlas/. Several regions had no clear consensus definition in the literature and therefore required specific delineation in this atlas. We have included precise descriptions of these regions in Supplementary Material, along with Supplementary Table 2 detailing the landmarks used to define the parcellation.

### Evaluation of parcel-level accuracy

**Figure 2:**
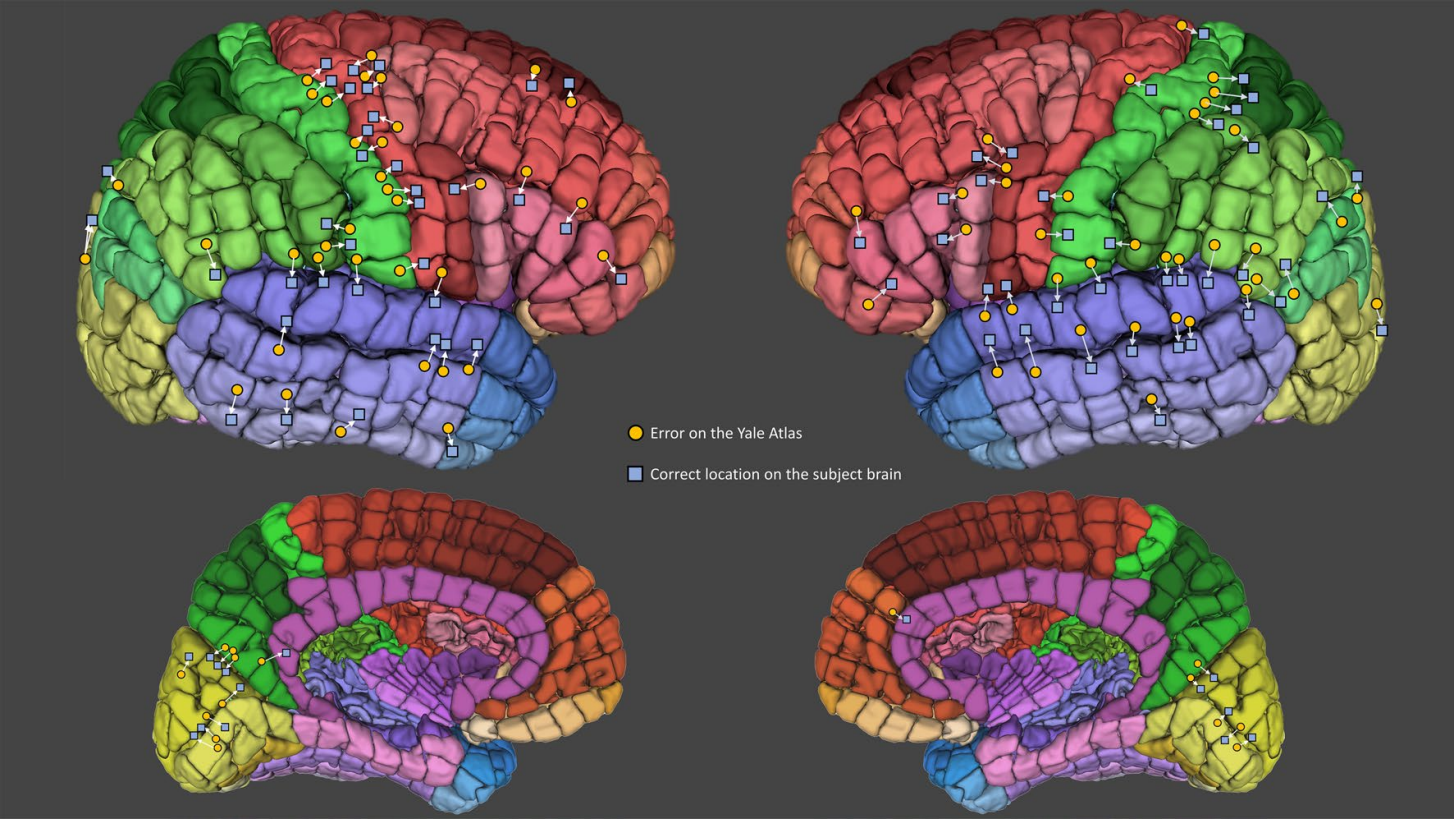
Error locations on the Yale Brain Atlas in a cohort of 25 patients with intracranial electrodes registered to the Atlas. Of the 3866 electrode contacts in the 25 subjects there were 93 which were localized to the incorrect parcel. Localization errors often clustered around certain anatomical landmarks, most commonly the central sulcus, Sylvian fissure and superior temporal sulcus.

There were 3866 electrode contacts in the 25 subjects (mean = 155/patient) with an approximately equal proportion of left-hemisphere to right-hemisphere contacts (1.05:1). These contacts were checked for anatomical accuracy. There were 93 electrode contacts that were in the incorrect parcel, gyrus, or lobe, indicating a localization accuracy of 97.6% (Fig. 2). The most common error localizations were to the S (somatosensory 14/93, 15.1%), T1 (superior temporal 6/93, 6.45%) and P4 (superior parietal 4 6/93, 6.45%) gyri, while the most common sulci and fissures across which contacts were erroneously localized were the central sulcus (12/93, 12.9%), Sylvian fissure (10/93, 10.8%), and superior temporal sulcus (8/93, 8.6%). The data from the accuracy evaluation study is made available under ‘YBA_study_data_1’ at https://www.nitrc.org/frs/?group_id=1532.

### Cohort atlas localization

**Figure 3:**
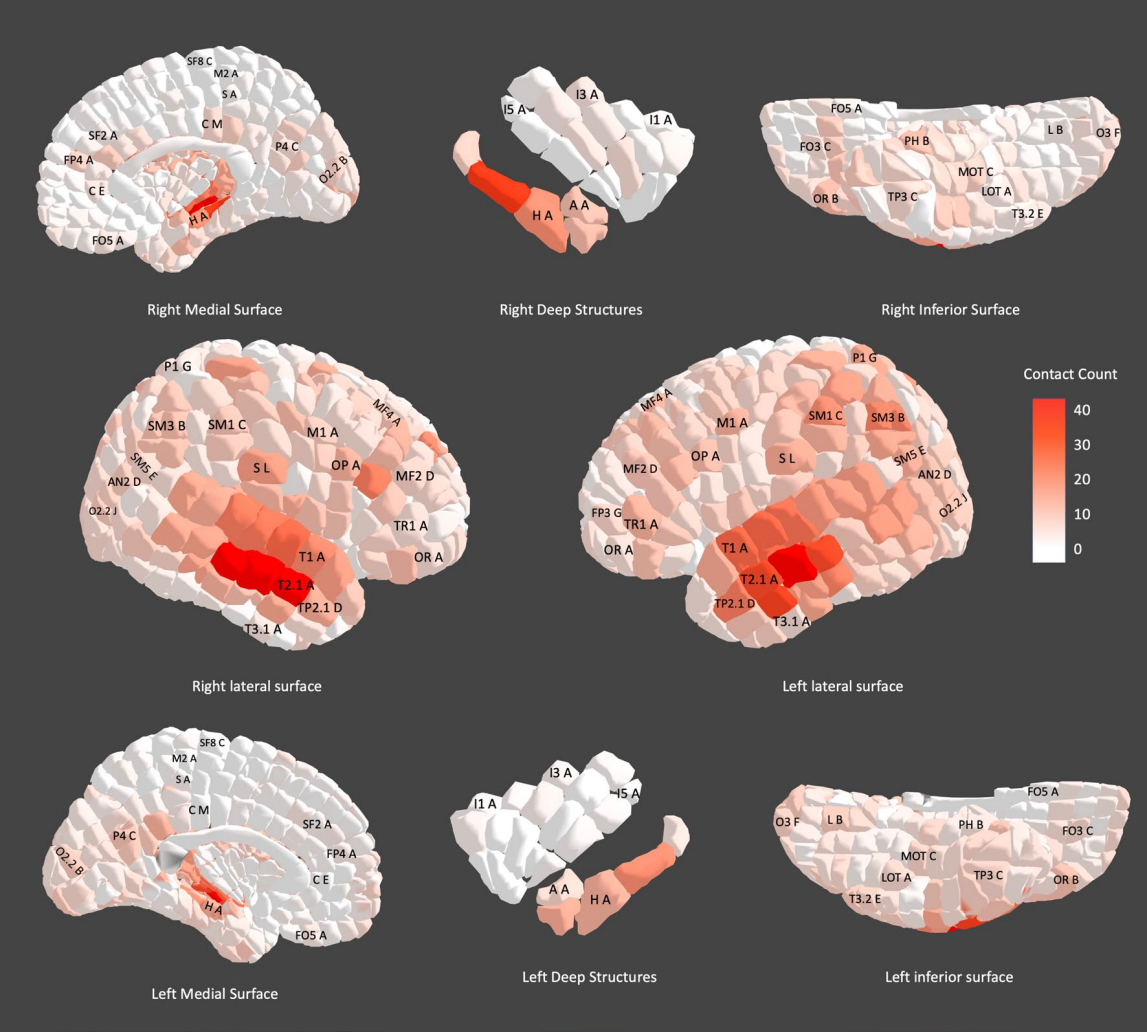
Electrode contact distribution across the cohort. We have used a new graphical coloring scheme with a spectrum of red-to-white to represent the density of electrode contact coverage in each parcel. Red represents a higher sampling frequency and white represents a lower sampling frequency. A single parcel with high coverage is labelled in each gyrus or pair of gyri for illustration of the atlas nomenclature.

Figure 3 shows the spatial distribution of electrode contacts across the cohort and the number of contacts per parcel. In terms of coverage, 573 out of 690 parcels had one or more contacts (82.3%). The mean number of contacts per parcel was 5.34, the median number of contacts per parcel was four and the range was 0–42 contacts per parcel. The parcels with the greatest coverage across the cohort were the R_T2.1_A (right superior middle temporal gyrus A, 42 contacts), R_T2.1_B (right superior middle temporal gyrus B, 42 contacts) and L_T2.1_B (left superior middle temporal gyrus B, 41 contacts). The regions with greater coverage were the lateral and medial temporal lobes bilaterally. The regions with the least coverage were the medial frontal lobe, the medial parietal lobe, and the insula. The data from the cohort localization study is made available under ‘YBA_study_data_1’ at https://www.nitrc.org/frs/?group_id=1532.

## Discussion

We have constructed a cortical atlas consisting of 690 parcels, each defined in relation to conserved neuroanatomical landmarks and with precise anatomical detail. The YBA provides a parcellation and nomenclature for brain localization to the nearest centimeter. There was a high degree of accuracy in individual-to-atlas registration based on the anatomical localization of intracranial electrodes, suggesting that the one-square centimeter parcellation is appropriate and useful for localizing high resolution data. When applied to clinical data the YBA showed utility for characterising the anatomical distribution of intracranial electrodes across a small cohort of subjects and highlighted the increased sampling of certain regions of the brain, which is an important step in many clinical studies to characterize bias and what effect this may have on results.

In our epilepsy surgery practice we noted a need to standardize anatomical brain localization to facilitate multimodal data fusion. Our solution, the YBA, was derived from the 40-year experience of a neurosurgeon in clinical epilepsy surgery (D.S.). Epilepsy surgery has enabled pioneering advances in applied neuroscience through the unique opportunity for collecting brain data in-vivo^20,21^. The unit of localization in epilepsy surgery is the icEEG electrode contact; either in a subdural electrode placed on the brain surface, or a depth electrode which passes through brain tissue. Electrode contacts on subdural or depth electrodes are typically spaced one-centimeter apart, and sample electrical activity from a small volume of surrounding tissue^19^. These electrodes are used to identify the region of the brain that produces seizures to guide precise treatment to the nearest centimeter of tissue; whether resection (surgical removal) or modulation (electrical stimulation treatment). Stimulation of these electrodes may also be used to identify regions of critical cortical function to guide surgery for epilepsy and for brain tumors so that there is no disruption of these regions, thereby ensuring the best treatment outcomes^22,23^. Locations of function and seizure characteristics, revealed through the chronic placement of intracranial electrodes, are often communicated based on common neuroanatomy. This is consistent with localization across the clinical and research neurosciences and has also been the method of localization in a number of popular brain atlases^14,16,24^. Present day unparcellated neuroanatomical localization may introduce regional ambiguity and may reduce the precision of the data. Communication of sub-gyral regions is desirable but poses a challenge due to individual variability in the gyral-sulcal pattern^25^. The YBA has been refined for accurate, sub-gyral localization according to the anatomical sulco-gyral pattern of the human brain and it has applicability across the neurosciences where precise, systematic localization of data across individuals is required.

There are several approaches to brain parcellation that all carry utility for different applications. Boundary mapping based on local features may segregate the brain according to gross anatomy, cytoarchitecture and myelin density, while connectivity-based approaches use global brain features such as functional connectivity and white matter connectivity^26,27^. One benefit of local boundary mapping is that it may offer a more intuitive regional segregation. Many prior studies have utilized data-driven, local boundary mapping approaches to parcellation, for instance using resting-state fMRI to identify cortical parcels based on changes in functional connectivity patterns^28,29^. These methods produced parcellations that showed homogeneity with known brain network maps, as well as with architectonically derived maps, suggesting that a true biological feature was identified. Boundary mapping based on cytoarchitecture is another local feature mapping method that began with Korbinian Brodmann’s seminal work, and has continued to develop with the advent of the Julich Brain recently^30,31^. The Destrieux atlas is a local, boundary-based atlas created using gross neuroanatomical boundaries including sulci and fissures^14^. While the approach lends itself to usability, since gross neuroanatomy is a widely recognized language for brain localization, it could be enhanced with precise sub-gyral localization, which may be required in many studies.

An alternative approach to boundary mapping based on local features is connectivity mapping using global features^2^. This approach is grounded in our understanding that rather than discrete locations being responsible for key neurologic functions, it is the distributed network architecture of the brain that provides the basis for neurologic function^32,33^. The authors of the Human Brainnetome Atlas used diffusion magnetic resonance imaging (MRI) and resting state functional MRI (fMRI) to identify anatomical and functional connectivity between brain regions to create a parcellation with 246 regions^4^. The authors of the Yeo Atlas used resting state fMRI alone to characterize 17 cortical networks^5^. These studies provided valuable insights into network organization of the human brain and offer valuable atlas tools for studies in which localization correlated with underlying networks and functional architecture is required. The HCP-MM1 (Glasser) atlas provides a multi-modal, data-driven parcellation based on consensus between measures of architecture, function, connectivity, and topography^3^. It has been applied successfully in many studies, including in the analysis of structural connectivity in specific white matter tracts^34,35^, as a cortical prior for the creation of new atlases^36^, and for localizing and assessing connectomic data^37^. It is an excellent parcellation for localizing data according to robust, biologically defined parcels. Its usefulness could be augmented by an anatomically defined parcellation for precise, sub-gyral localization. For example, where studies require sub-gyral parcellation of Area 4 of the HCP-MM1, the primary motor cortex, to localize specific functions within the gyrus.

The aim of our boundary mapping approach was to ensure a transparent and understandable regional segregation by using common anatomical brain features to define our parcellation. We identified landmarks based on a consensus designation from three resources and generated areal cortical boundaries using these landmarks as a guide. The parcellation was ultimately guided by boundaries that were clinically useful in neurosurgical practice, based on decades of clinical experience. Conceptually our atlas was created as a tool to localize multimodal data to facilitate data exposition, exploration, and analysis. A key strength of the YBA lies in its high resolution parcellation and systematic regionalization, which lends itself to modern data science approaches since parcels are approximately homogeneous in surface area and can be further partitioned if even higher resolution parcellation is needed. There are other atlases available that offer a high-resolution parcellation, including the Shen Atlas, a resting-state fMRI atlas with up to 300 discrete nodes^38^, the Local–Global Parcellation based on fMRI data with up to 1000 nodes^6^, and the Connectome Mapper with up to 1015 nodes^39^. These and other approaches offer granularity but could be refined for studies where a practical, systematic parcellation that is constrained by the underlying sulco-gyral pattern is necessary. The YBA offers this functionality, along with useability in clinical practice.

Our accuracy test of atlas registration used intracranial electrode contacts to provide a substrate for anatomical localization. We noted that registration errors, likely resulting from a difference between the anatomy of the MNI152 averaged brain and the anatomy of individual subjects, commonly localized to three major areas: the central sulcus, the Sylvian fissure, and the superior temporal sulcus. A major strength of our accuracy study approach is the discrete localization of electrode contacts to specific structures in the brain, enabling direct anatomical comparison. A potential weakness of our approach results from the lack of standardized electrode sampling between patients. We noted that certain regions of the cortex, such as the superior and middle temporal gyri, as well as the supramarginal gyrus, were sampled at high frequencies across the cohort due to their common involvement in epilepsy networks. This regional sampling bias is likely to have introduced regional bias into our localization error rate. With a larger sample size consisting of patients with a variety of epilepsies, we could control for this factor and thereby achieve a more accurate representation of topographic cortical variation between individuals. Another limitation of our approach is due to the selection bias of the study. We selected subjects with no gross cortical malformations or anatomical distortion to ensure maximal registration accuracy. We also selected subjects independently of age or developmental stage. Our approach may be less accurate on grossly deformed brains, and in certain age ranges. However, the approach we used is identical to our robust clinical image registration protocol using tensor and B-spline methods, which we have used for 20 years in many surgical patients, including those with large anatomical distortions. We have found clinically useful registration accuracy using this approach. A similar algorithm, the IRTK algorithm, demonstrated excellent performance in comparison to other registration approaches^40^. The study also found that the performance of the algorithms was relatively unaffected by the study population, indicating a high degree of generalizability, which is important for application to our data. We will explore the effect of anatomical distortion on our registration accuracy and on the useability of the atlas in a future work.

To-date we have used a pipeline in BioImage Suite, an open-source software package for medical image processing, to apply the YBA to epilepsy surgery data^41^. We offer the Atlas as a NIfTI file for users who wish to implement it in their own workflows (https://www.nitrc.org/projects/yale_atlas_2021/). We are working to provide the Atlas as a database tool through an online platform (https://yalebrainatlas.github.io/YaleBrainAtlas/), where users can upload their own data in MNI space, and we can provide localizations for this data on the YBA to assist with precise communication. Functional, structural, metabolic and electrophysiology data will be registered to the common atlas space to facilitate data aggregation and anatomical localization. The high resolution and standardization of the parcellation lends itself to computational analysis, with the unit of comparison being the parcel, gyrus, region, or lobe depending on the needs of the analysis. Soon we intend to compute the YBA in native patient space, thereby foregoing the need to register and transform data from the patient to the MNI152 brain with the inaccuracies this may introduce. This may aid the localization and communication of individual data for case reports and case studies in research as well as in case discussions in clinical practice. Clinically the Atlas may also provide a tool for surgical planning, for instance planning stereo-EEG studies which use depth electrodes to sample deep areas of the brain, where information about cortical surface parcels and deep parcels may be useful to provide entry and target sites. Stereo-EEG electrode entry sites are typically spaced a minimum of one-centimeter apart, and the YBA nomenclature provides a practical tool to designate where each electrode enters the brain and the name of its target. The YBA is granular enough to provide discrete localization for two stereo-EEG electrodes with separate points of entry on the cortical surface, or for multiple stereo-EEG contacts within the same electrode that are passing along the cortical surface, for instance electrodes that are placed obliquely into the insula.

We are also working on providing the YBA as an educational pedagogic tool for teaching structure–function relationships. The high resolution, and intuitive organization into anatomically defined subregions offers value since most educational programs link structure to function using topographic relationships^42–44^. It can effectively augment these workflows by providing precise, systematic localization where pathology in sub-gyral structures is known to cause discrete syndromes, for instance in Gerstmann syndrome^8^, the focal epilepsies^45–48^, as well as focal functional deficits following stroke^49^.

Several areas in the YBA may be of particular interest for linking function with structure. Functional localization in the brain has been widely reported using Brodmann areas, common neuroanatomy, and functional designations, including in language, motor and sensory regions^7,22,50–52^. The lateral occipital complex is a region involved in object recognition, and is found in the occipital lobe inferior to the lateral occipital sulcus, and towards the posterior end of the fusiform gyrus^53^. This can be sub-parcellated to an area approximately consisting of MOT_H, LOT_E, O3_I, and O3_H in the YBA, enabling finer parcellation, and more precise communication. The primary motor cortex, precentral gyrus, or Brodmann area 4, is a region of cortex involved in motor control^54^. It is divided into parcels M1_A through M1_D and M2_A through M2_K in the YBA, enabling precise localization of specific motor functions, as in the motor homunculus, and communication of these areas. The inferior frontal gyrus contains pars opercularis and pars triangularis, or Brodmann areas 44 and 45, which correlates with Broca’s speech area^55^. The region is sub-parcellated in the YBA into OP_A through OP_C, and TR1, TR2, and TR3_A and _B. Application of the YBA to these different functional localization problems enables precise localization and communication of regions of function within these gyri, thereby ensuring reproducibility. We are also working to incorporate information on white matter tracts between parcels in the YBA. This information would assist in connectivity analyses based on fMRI data or on electrophysiology by providing an accurate representation of structural connectivity between regions^56,57^. The standardized parcel sizing may allow for future sub-parcellations of the existing YBA, for instance if new electrodes with smaller inter-contact distances become widely used. Any new sub-parcellation will have to be carefully balanced with the constraints of image registration to maintain registration accuracy.

We have presented a novel whole-brain atlas of the cortex which is systematically parcellated into 690 regions based on common neuroanatomy. We have detailed our anatomical designations across the cortex based on a consensus between the literature, the MNI152 Symmetric brain and a small structural imaging dataset. We have also tested the accuracy and usefulness of one-square centimeter, parcel-based localization using a small cohort of subjects. The YBA is intended for application to any problem which requires precise, systematic localization and communication of information on the cortex, including structural, functional, and metabolic data among others.

## Methods

### Atlas creation

We used three resources to build the YBA; the ICBM (MNI) 152 Nonlinear Symmetric atlas was the template brain, selected for its robust representation of normative population anatomy^58^. The second resource was Duvernoy’s Atlas of the Human Brain^59^, a widely-used visual reference atlas for labelling human brain anatomy according to international standards for anatomical terminology (or the Terminologia Anatomica)^60^. The third resource was a dataset of anatomically normal structural MRI scans from a cohort of 25 subjects who underwent evaluation for epilepsy surgery, providing a reasonable sample size to ensure the majority of parcels were covered.

**Figure 4:**
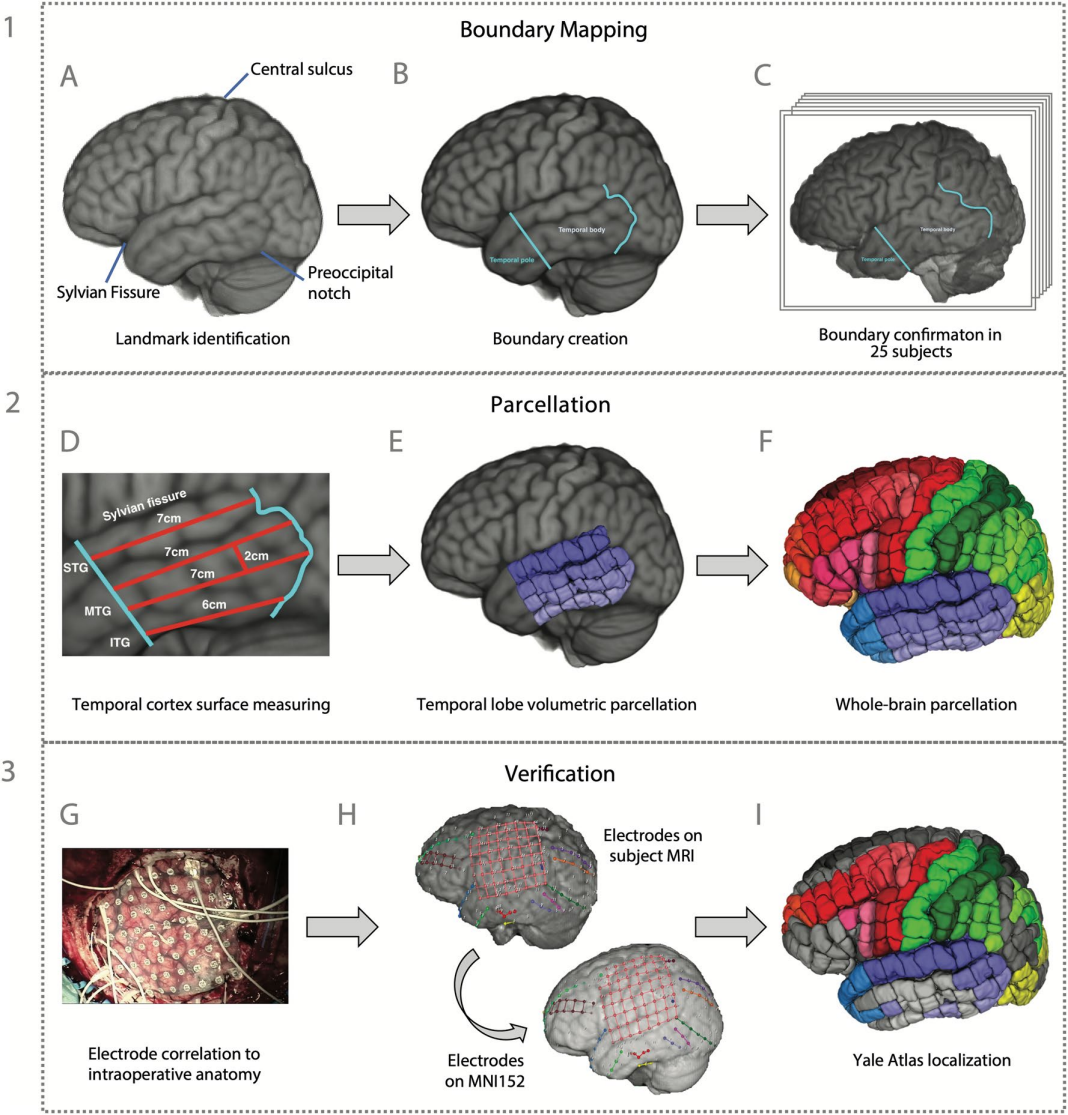
The workflow used to create the atlas and the methods used to assess the accuracy and usefulness of the YBA for clinical application. The workflow is demonstrated in (**A**–**F**) using the temporal lobe for illustration. (**A**) The ICBM (MNI) 152 template was labelled anatomically using designations in Duvernoy’s Atlas of the Human Brain. (**B**) Boundaries were created for the Yale Brain Atlas (YBA) between major anatomical landmarks to distinguish the lobar and sub-lobar regions. (**C**) The YBA anatomical boundaries were checked against 25 individual MRI scans to assess validity. (**D**) Cortical surface measures were used between major atlas boundaries to identify the number of parcels in each region. The middle temporal gyrus was approximately two-centimeters in width, so it was split in two to maintain the one-square centimeter parcellation. (**E**) The temporal body was parcellated into superior temporal, middle temporal, and inferior temporal regions. (**F**) The parcellation was continued across the entire MNI152 template to create the YBA. (**G**) The first step in evaluation anatomical accuracy was to use intraoperative images as a ‘ground truth’ for electrode contact localization and relate the contacts to topographical neuroanatomy. (**H**) MR imaging for each patient was compared with that patient’s ground truth, intraoperative image for accuracy. Once the electrode localizations were verified as accurate, the electrodes were transformed to the MNI152 brain. (**I**) The YBA parcellation was labelled according to the electrode locations to facilitate precise, cohort-wide localization.

We first identified common neuroanatomical landmarks using the MNI152 template as a visual reference and using Duvernoy’s atlas to label the anatomical landmarks (Fig. 4). Sulci and gyri were identified in 2D MRI space using the 2D windows in ITK-SNAP, and this was correlated with the 3D volume in FSLeyes. We then checked these landmarks against the MRI anatomy of the cohort to assess the real-world validity of our designations. If a labelled structure was visible on the subjects’ MRI scans, we considered this a valid anatomical landmark. Wider literature was used where these three resources did not provide enough anatomical detail to guide our designations, and we have detailed this where relevant. The YBA boundaries were created as intersections of these common anatomical landmarks using the image segmentation module in ITK-SNAP^61^. Most of the atlas boundaries are widely recognized, consensus designations, for instance the Sylvian fissure as the separation of the temporal, frontal, and parietal lobes. Some of the boundaries were defined specifically for this atlas due to the lack of a consensus in the literature regarding the precise definition of cortical areas and gyri, for example the frontal and temporal poles.

Once the regional cortical boundaries were created, we parcellated the cortex in 3D volume space to reflect the underlying sulci and gyri of the MNI152 brain, and to approximate one centimeter along the surface of each gyrus. The depth of each parcel was equal to the depth of the cortex and was approximated based on the contrast at the gray-white junction. The one-square centimeter parcellation was selected to accommodate the high-resolution labelling of intracranial surface electrodes placed on the brain for the surgical evaluation of epilepsy, where the inter-contact spacing is one centimeter, and to accommodate the typical minimum distance between insertion sites of intracranial depth electrodes, which are placed in the brain to access deep structures or to avoid opening the skull surgically. This spacing allows us to incorporate information captured by these sensors at the one centimeter acquired resolution. This is important since data procured from electrodes provides a spatial reference for treatment decisions and for findings in functional and structural imaging studies, including tumors and dysplastic lesions, which are typically localized based on their proximity to seizure activity and therefore electrode locations. The size of each parcel was measured as an image annotation in the 2D windows of ITK-SNAP and checked using the 3D viewer in FSLeyes, and the surface viewer in BioImage Suite. The first measurement was the total length of the gyrus from one major anatomical boundary to the next. The gyrus was then divided into parcels of equal length along the gyrus in a manner that most accurately approximated one-centimeter per parcel. The width of each parcel was equal to the width of the gyrus in nearly all cases, since this closely approximated one-centimeter. Where the width of the gyrus was greater than 1.5 cm, the gyrus was split in two and two parallel rows of parcels were created. The polygon and paintbrush segmentation tools in ITK-SNAP were used to create the parcellation in the axial, coronal, and sagittal windows^61^. This was then rendered as a 3D volume to check the result. This process was repeated to create all the parcels. A video demonstration was used for additional guidance during the segmentation of the hippocampus and amygdala^62^. The parcellation of one complete hemisphere was flipped and mirrored using the Convert3d module of ITK-SNAP to create a symmetric parcellation on the contralateral side.

### Nomenclature

A standardized nomenclature is offered with the YBA. Each gyrus has a unique identifier, based on common neuroanatomical nomenclature. Most have a letter code followed by a number, indicating the position of a gyrus within a grouping, for instance P1 as the anterior gyrus of the superior parietal grouping, and P4 as the posterior gyrus. There is an additional feature included in some gyri. Where a single gyrus is too large to encompass a one-centimeter parcel width, a decimal code is used, for instance the middle occipital gyrus (O2) has been split into superior middle (O2.1) and inferior middle (O2.2) occipital sub-gyri. Part of the cingulate gyrus has a decimal code where it is expanded in width to incorporate two parcels (C_N.1 and C_N.2). Across the YBA, parcels within a gyrus are named sequentially by letter, with the ‘A’ parcel denoting the beginning of a gyrus and each centimeter increment labelled with the subsequent letter. The sequence continues until a fixed regional boundary, such as a sulcus, or a key anatomical landmark is reached. To aid visualization, each lobe has a unique color designation and gyri within a region are colored to reflect their position within the region. The ‘first’ gyrus (anterior-most or superior-most) has the darkest shade, while the ‘Last’ gyrus has the lightest shade.

### Subjects

Twenty-five consecutive subjects were selected for accuracy evaluation from a large epilepsy surgery cohort. The cohort consisted of 14 females (14/25, 56%) and had a mean age of 36.7 years ± 12.5, range 8–60 years. The study was conducted with approval from the Yale Institutional Review Board under protocol number 2000021736. The subjects provided written informed consent for the use of their data in the study. The study was conducted in accordance with the relevant guidelines and regulations.

### Data processing and analysis

We used BioImageSuite for data visualization and image verification^41^. We used the biswebnode command-line tools for image registration and data processing (https://www.npmjs.com/package/biswebnode). Biswebnode uses a tensor and B-spline based non-linear registration method with normalized mutual information as the similarity metric.

Each subject had intracranial electrodes placed in the brain for epilepsy evaluation along with two whole-brain MRI scans; one performed preoperatively, and one postoperatively, and one postoperative computed tomography (CT) scan. The preoperative MRI was used for atlas image registration due to the higher quality of the image (3 T vs 1.5 T), and the lack of postoperative artefact. The preoperative MRI was performed using a Siemens Magnetom 3.0 T scanner (Siemens, Erlangen, Germany), sequence: MPRAGE, parameters: TR/TE = 1900/3 ms, flip angle = 9°, TI = 900 ms, average = 1, slice thickness = 1 mm, matrix = 256 × 256, FOV = 240 × 216. All images were checked for quality as part of our clinical protocol. The postoperative CT scan was used to label the intracranial electrode contacts in 3D space using the Electrode Editor of BioImage Suite. The electrode contacts were then transformed to the skull-stripped postoperative MRI and then the skull-stripped preoperative (3 T) MRI using a concatenation of four discrete transformations based on control point spacing (CPS)^63^, which were:

1. A non-linear B-spline transformation at resolution CPS.
2. A non-linear B-spline transformation at resolution CPS*2.
3. A non-linear B-spline transformation at resolution CPS*4.
4. Affine transformation to normalize for overall position and shape.

These steps were used to minimize registration error and postoperative artefact and are routinely undertaken for all patients who undergo intracranial EEG monitoring at our center. Following the above steps, the preoperative MR images were non-linearly registered to the MNI152 space for this study. The electrode contacts were transformed to the MNI space for anatomical correlation between the individual and atlas spaces.

To evaluate the accuracy of individual-to-atlas registration, and to test our hypothesis that the one-square centimeter parcellation was appropriate given the anatomical nuances of individual brains, we visually related electrode contacts to key landmarks between the MNI and individual (pre-transformed) brains. Electrode contacts which were localized to a different structure between the YBA and individual brains were labelled as outliers based on their incorrect parcel, gyral or lobar designation. We then used the YBA to localize, visualize and communicate the cohort-wide electrode distribution to characterize sampling biases in our surgical program. This is an important limitation in many studies based around surgical epilepsy data where some regions may have increased sampling coverage by intracranial electrodes, thereby creating false-positive associations.

### Ethics statement

The study was conducted with approval from the Yale Institutional Review Board under protocol number 2000021736. The subjects provided written informed consent for the use of their data in the study.

## Supplementary Information

Supplementary Information.

## Data Availability

The Atlas can be downloaded from https://www.nitrc.org/frs/?group_id=1532 and it can be viewed at https://yalebrainatlas.github.io/YaleBrainAtlas/. A table of the gyri and parcels of the Atlas is provided as Supplementary Table 1 in Supplementary Material. A table of the landmarks used to define the Atlas is provided as Supplementary Table 2 in Supplementary Material. A summary of the regions that required specific designations for the atlas, along with relevant literature, is provided at the end of Supplementary Material. The data from the accuracy evaluation study, the cohort localization study along with the code and data used to generate Fig. 4 are provided under ‘YBA_study_data_1’ at https://www.nitrc.org/frs/?group_id=1532.
