# Supplementary material for: High-resolution cortical parcellation based on conserved brain landmarks for localization of multimodal data to the nearest centimeter
Source: Sci Rep. 2022 Nov 5;12:18778. doi: 10.1038/s41598-022-21543-3 (PMC9637135; doi:10.1038/s41598-022-21543-3)
Supplement: Supplementary file 1 — Supplementary Information. [file 41598_2022_21543_MOESM1_ESM.docx]

Supplementary Table 1. The gyri of the atlas along with an accompanying code for each and the number of parcels in the gyrus. There are 690 parcels and 144 gyri within 34 discrete, sub-lobar regions, including the temporal pole and frontal orbital cortex, and 10 lobes. The designations are identical for the two hemispheres. The anatomical details of each gyrus are provided in the last column, where appropriate.

| ID | Gyrus | Code | Parcels | Anatomical Details |
| --- | --- | --- | --- | --- |
| 1 | Superior temporal pole | TP1 | 6 | The anterior continuation of the superior temporal gyrus. Beginning posterior-lateral at the border of the temporal pole and curving medially to the entorhinal cortex, or semilunar gyrus of the parahippocampus. |
| 2 | Superior middle temporal pole | TP2.1 | 4 | The anterior continuation of the superior aspect of the middle temporal gyrus, curving medially to the entorhinal cortex, or gyrus ambiens of the parahippocampus. |
| 3 | Inferior middle temporal pole | TP2.2 | 4 | The anterior continuation of the inferior aspect of the middle temporal gyrus curving medially to the entorhinal cortex, or gyrus ambiens of the parahippocampus. |
| 4 | Inferior temporal pole | TP3 | 4 | The anterior continuation of the inferior temporal gyrus. Ends medially at the anterior point of the rhinal sulcus, in front of the anterior transverse collateral sulcus. |
| 5 | Superior temporal gyrus | T1 | 7 | The cortex above the superior temporal sulcus. Terminates posteriorly at the supramarginal gyrus, below the ascending ramus of the Sylvian fissure. |
| 6 | Superior middle temporal gyrus | T2.1 | 7 | The superior ~ 1cm width of cortex of the middle temporal gyrus, below the superior temporal sulcus and ending at the posterior temporal border. |
| 7 | Inferior middle temporal gyrus | T2.2 | 7 | The inferior ~ 1cm width of cortex in the middle temporal gyrus. Below the superior temporal sulcus and ending at the posterior temporal border. |
| 8 | Lateral inferior temporal gyrus | T3.1 | 6 | The lateral portion of cortex below the inferior temporal sulcus and ending posteriorly at the preoccipital notch. |
| 9 | Basal inferior temporal gyrus | T3.2 | 5 | The basal portion of the inferior temporal gyrus, lateral to the lateral occipitotemporal sulcus. |
| 10 | Superior occipital gyrus | O1 | 5 | The gyrus inferior to the parieto-occipital fissure in the occipital lobe and superior to the transverse occipital sulcus on the lateral surface. |
| 11 | Superior middle occipital gyrus | O2.1 | 5 | The superior aspect of the middle occipital gyrus, inferior to the transverse occipital sulcus and superior to the lateral occipital sulcus. |
| 12 | Inferior middle occipital gyrus | O2.2 | 11 | The inferior aspect of the middle occipital gyrus, inferior to the lateral occipital sulcus and superior to the inferior occipital sulcus laterally and the calcarine sulcus medially. |
| 13 | Inferior occipital gyrus | O3 | 11 | Begins medially behind the posterior parahippocampus as it meets the isthmus of the cingulate, and curves inferior to the calcarine and inferior occipital sulci to meet the preoccipital notch laterally. |
| 14 | Fourth occipital gyrus | O4 | 2 | The occipital gyrus in the basal occipital lobe, anterior to the occipital pole and bounded anteriorly by the posterior transverse collateral sulcus. |
| 15 | Superior angular gyrus | AN1 | 3 | The cortex inferior to the sulcus intermedius primus of Jensen. |
| 16 | Inferior angular gyrus | AN2 | 4 | Anterior and lateral to the superior occipital gyrus and extending posterior as a continuation of the middle temporal gyrus. |
| 17 | Supramarginal gyrus 1 | SM1 | 5 | The anterior portion of the supramarginal gyrus, posterior to the post-central sulcus and inferior to the intraparietal sulcus. |
| 18 | Supramarginal gyrus 2 | SM2 | 2 | A small nub of gyrus anterior to the parallel sulcus. |
| 19 | Supramarginal gyrus 3 | SM3 | 3 | The posterior part of the segment of the supramarginal gyrus that is anterior to the ascending ramus of the Sylvian fissure. |
| 20 | Supramarginal gyrus 4 | SM4 | 3 | The anterior part of the segment of the supramarginal gyrus that is posterior to the ascending ramus of the Sylvian fissure. |
| 21 | Supramarginal gyrus 5 | SM5 | 5 | The cortex superior and anterior to the sulcus intermedius primus of Jensen, extending posterior as a continuation of T1, inferior and posterior to the ascending ramus of the Sylvian fissure. |
| 22 | Superior parietal 1 | P1 | 7 | The anterior aspect of the superior parietal lobule, anteriorly bounded by the post-central sulcus and posteriorly by the transverse parietal sulcus. |
| 23 | Superior parietal 2 | P2 | 4 | Posterior to the transverse parietal sulcus and anterior to the anterior medial ramus of the intraparietal sulcus. |
| 24 | Superior parietal 3 | P3 | 6 | Between the P2 and P4 gyri, and superior to the posterior medial ramus of the intraparietal sulcus. |
| 25 | Superior parietal 4 | P4 | 7 | Superior to the superior occipital gyrus and abutting the parieto-occipital fissure medially to the isthmus of the cingulate gyrus. |
| 26 | Somatosensory | S | 13 | The postcentral cortex, anterior to the postcentral sulcus. |
| 27 | Anterior motor | M1 | 4 | A small segment of the inferior lateral motor cortex, posterior to pars opercularis and the precentral sulcus. |
| 28 | Posterior motor | M2 | 11 | The major segment of the motor cortex, anterior to the central sulcus and posterior to the precentral sulcus. |
| 29 | Superior frontal 1 | SF1 | 4 | The anterior segment of the superior frontal region, anteriorly bounded by the frontal pole and beginning medially at the cingulate sulcus. |
| 30 | Superior frontal 2 | SF2 | 4 | The second segment of the superior frontal region. |
| 31 | Superior frontal 3 | SF3 | 4 | The third segment of the superior frontal region. |
| 32 | Superior frontal 4 | SF4 | 4 | The fourth segment of the superior frontal region. |
| 33 | Superior frontal 5 | SF5 | 4 | The fifth segment of the superior frontal region. |
| 34 | Superior frontal 6 | SF6 | 4 | The sixth segment of the superior frontal region. |
| 35 | Superior frontal 7 | SF7 | 5 | The seventh segment of the superior frontal region. |
| 36 | Superior frontal 8 | SF8 | 4 | The posterior segment of the superior frontal gyrus, anterior to the precentral sulcus. |
| 37 | Middle frontal 1 | MF1 | 3 | The anterior segment of the middle frontal region inferior to the superior frontal sulcus and extending laterally to the inferior frontal sulcus. |
| 38 | Middle frontal 2 | MF2 | 4 | The second segment of the middle frontal gyrus, extending medially in a line with the second segment of pars triangularis. |
| 39 | Middle frontal 3 | MF3 | 4 | The third segment of the middle frontal gyrus, extending medially in a line with the third segment of pars triangularis. |
| 40 | Middle frontal 4 | MF4 | 4 | The fourth segment of the middle frontal gyrus, extending medially in a line with pars opercularis. |
| 41 | Middle frontal 5 | MF5 | 3 | The fifth segment of the middle frontal gyrus. |
| 42 | Middle frontal 6 | MF6 | 3 | The sixth segment of the middle frontal gyrus. |
| 43 | Middle frontal 7 | MF7 | 3 | The seventh segment of the middle frontal gyrus. |
| 44 | Middle frontal 8 | MF8 | 1 | The eighth segment of the middle frontal gyrus, anterior to the precentral sulcus. |
| 45 | Pars opercularis | OP | 3 | The posterior part of the inferior frontal gyrus, posterior to the vertical ramus of the Sylvian fissure and anterior to the precentral sulcus. |
| 46 | Anterior pars triangularis | TR1 | 2 | Posterior to the pars orbitalis and superior to the limen insulae. |
| 47 | Middle pars triangularis | TR2 | 2 | The middle segment of pars triangularis. |
| 48 | Posterior pars triangularis | TR3 | 2 | Anterior to the vertical ramus of the Sylvian fissure. |
| 49 | Pars orbitalis | OR | 3 | The anterior segment of the inferior frontal gyrus. |
| 50 | Frontal pole 1 | FP1 | 8 | The first of the inferior transverse frontopolar gyri, beginning medially in front of the subcallosal gyrus and superior to the susorbital sulcus and extending laterally just above the frontomarginal sulcus. |
| 51 | Frontal pole 2 | FP2 | 4 | The second of the inferior transverse frontopolar gyri. |
| 52 | Frontal pole 3 | FP3 | 7 | The first of the superior transverse frontopolar gyri. |
| 53 | Frontal pole 4 | FP4 | 6 | The second of the superior transverse frontopolar gyri and the posterior gyrus of the frontal pole. |
| 54 | Frontal orbital 1 | FO1 | 4 | The anterior segment of the inferior surface of the frontal lobe, bounded anteriorly by the frontomarginal sulcus, beginning medially at the gyrus rectus and extending laterally to pars orbitalis. |
| 55 | Frontal orbital 2 | FO2 | 5 | The second segment of the frontal orbital region, posterior to the first and at the anterior aspect of the H-shaped orbital sulcus. |
| 56 | Frontal orbital 3 | FO3 | 4 | The third segment of the frontal orbital region at the posterior aspect of the H-shaped orbital sulcus. |
| 57 | Frontal orbital 4 | FO4 | 4 | The fourth segment of the frontal orbital region, bounded posteriorly by the arcuate orbital sulcus |
| 58 | Frontal orbital 5 | FO5 | 5 | The fifth segment of the frontal orbital region, consisting mostly of the posterior orbital gyrus. |
| 59 | Frontal orbital 6 | FO6 | 3 | The posterior aspect of the frontal orbital region, consisting mostly of the posterior orbital gyrus. |
| 60 | Lateral occipitotemporal gyrus | LOT | 5 | Forms the lateral part of the fusiform gyrus. Medial to the lateral occipitotemporal sulcus. |
| 61 | Medial occipitotemporal gyrus | MOT | 8 | Forms the medial part of the fusiform gyrus. Lateral to the collateral sulcus. |
| 62 | Parahippocampal gyrus | PH | 7 | Inferior to the hippocampus and amygdala and medial to the collateral sulcus. Meets the inferior part of the temporal pole anteriorly. |
| 63 | Lingual gyrus | L | 3 | The posterior continuation of the parahippocampal gyrus into the occipital lobe as it meets the isthmus of the cingulate gyrus. |
| 64 | Cingulate gyrus | C | 19 | A large gyrus wrapped around the corpus callosum, beginning anteriorly at the subcallosal area and extending posteriorly to meet the tail of the hippocampus. |
| 65 | Hippocampus | H | 3 | Split into pes, body and tail. |
| 66 | Amygdala | A | 2 | Anterior to the pes hippocampus. Split into superior and inferior parts to incorporate the major nuclei. |
| 67 | Anterior short insular gyrus | I1 | 3 | The first of the short insular gyri. |
| 68 | Posterior short insular gyrus | I2 | 3 | The second of the short insular gyri. Anterior to the central sulcus of the insula. |
| 69 | Anterior long insular gyrus | I3 | 3 | The first of the long insular gyri. Posterior to the central sulcus of the insula. |
| 70 | Middle long insular gyrus | I4 | 4 | The second of the long insular gyri. |
| 71 | Posterior long insular gyrus | I5 | 4 | The posterior gyrus of the insula. |

Supplementary Table 2. The landmarks used in the Atlas, defined using descriptions and labels in Duvernoy’s Atlas of the Human Brain, the visual anatomy of the MNI 152 Symmetric brain template and the MRI anatomy of 25 individual subjects.

| **Landmark** | **Description and significance** |
| --- | --- |
| Sylvian fissure | A deep fissure separating the temporal lobe from the frontal and parietal lobes. The transition point between the horizontal ramus and ascending ramus forms an important landmark for the boundaries of the temporal, occipital, and parietal lobes. |
| Superior temporal sulcus | Demarcates the boundary between the superior and middle temporal gyri. Its continuation posteriorly, as the sulcus intermedius primus of Jensen, indicates the border between the angular and supramarginal gyri in the inferior parietal lobe. |
| Pars opercularis | A straight line running from the vertical ramus of the Sylvian fissure, anterior to pars opercularis, to the anterior border of the amygdala defines the temporal pole-temporal body boundary. |
| Preoccipital notch | Forms the infero-lateral point of the boundary between the temporal lobe and the occipital lobe. The boundary begins laterally below the ascending ramus of the Sylvian fissure and curves inferior and posterior, following the anterior occipital sulcus, to the notch and then medially to the isthmus of the cingulate gyrus. |
| Parieto-occipital fissure | A deep fissure, prominent on the medial surface of the brian beginning anterior at a meeting point with the calcarine sulcus and separating the occipital lobe from the parietal lobe. |
| Intraparietal sulcus | Separates the superior parietal lobule from the inferior parietal lobule. Begins anterior at the postcentral sulcus and extends posterior to the occipital lobe. |
| Central sulcus | Separates the motor cortex of the frontal lobe from the somatosensory cortex of the parietal lobe. |
| Superior frontal sulcus | Separates the middle and superior frontal gyri. Extends anterior into the frontal pole as the frontomarginal sulcus, which separates the orbitofrontal region from the pole. |
| Inferior frontal sulcus | Separates the middle and inferior frontal gyri. |
| Gyrus rectus | Medially in the orbitofrontal cortex, separates the frontal pole medially from the orbitofrontal cortex. Laterally bounded by the straight sulcus and medially by the susorbital sulcus. |
| Collateral sulcus | Abuts the parahippocampal gyrus and ends posteriorly at the posterior transverse collateral sulcus, where the occipitotemporal gyri end and the fourth occipital gyrus begins. |
| Lateral occipitotemporal sulcus | Separates the lateral occipitotemporal gyrus from the inferior temporal gyrus. |
| Cingulate sulcus | Abuts the cingulate gyrus. The ascending ramus demarcates the boundary between the superior parietal lobule and the postcentral gyrus. |
| Central sulcus of the insula | Separates the long insular gyri from the short insular gyri. |
| Corpus callosum | The interhemispheric white matter bundles. The anterior commissure forms the medial point of a straight line running over the superior frontal sulcus that separates the frontal pole and frontal body. |

**Specific parcellation of atlas regions**

Some atlas regions had consensus designations in the literature, for instance the division of the hippocampus into the pes, body and tail, in the anterior-posterior axis ^1–3^. However, other regions required a thorough literature search along with some specific anatomical designations for this atlas. The frontal pole is an example, where the posterior border of the frontal pole begins anterior to the termination of the superior frontal sulcus ^4^. However, the sulcus can terminate variably between individuals and therefore we elected to define the pole in relation to a straight line between two robust landmarks: the anterior commissure and pars triangularis. The temporal pole is another example of a region with many different definitions in the literature ^5^. Our designation is in practical agreement with the surgical anatomy of this region for temporal pole resections and outlines the temporal pole as a distinct portion of the cortex ^6,7^. In the parietal lobe, we used Duvernoy’s definition of the angular and supramarginal gyri as continuations of the middle and superior temporal gyri respectively, separated by the sulcus intermedius primus of Jensen.

1. Berron, D. *et al.* A protocol for manual segmentation of medial temporal lobe subregions in 7 Tesla MRI. *NeuroImage. Clin.* **15**, 466–482 (2017).

2. Duvernoy, H. M. *The human hippocampus: functional anatomy, vascularization and serial sections with MRI*. (Springer Science & Business Media, 2005).

3. Daugherty, A. M., Bender, A. R., Raz, N. & Ofen, N. Age differences in hippocampal subfield volumes from childhood to late adulthood. *Hippocampus* **26**, 220–228 (2016).

4. Bludau, S. *et al.* Cytoarchitecture, probability maps and functions of the human frontal pole. *Neuroimage* **93**, 260–275 (2014).

5. Chabardès, S., Kahane, P., Minotti, L., Hoffmann, D. & Benabid, A.-L. Anatomy of the temporal pole region. *Epileptic Disord.* **4**, S9–S16 (2002).

6. Spencer, D. D., Spencer, S. S., Mattson, R. H., Williamson, P. D. & Novelly, R. A. Access to the Posterior Medial Temporal Lobe Structures in the Surgical Treatment of Temporal Lobe Epilepsy. *Neurosurgery* **15**, 667–671 (1984).

7. Helmstaedter, C. *et al.* Differential effects of temporal pole resection with amygdalohippocampectomy versus selective amygdalohippocampectomy on material-specific memory in patients with mesial temporal lobe epilepsy. *Epilepsia* **49**, 88–97 (2008).
